# Leptin acts on mesenchymal stem cells to promote chemoresistance in osteosarcoma cells

**DOI:** 10.18632/aging.103027

**Published:** 2020-04-14

**Authors:** Helin Feng, Qianqian Zhang, Yi Zhao, Lili Zhao, Baoen Shan

**Affiliations:** 1 Department of Orthopedics, The Fourth Hospital of Hebei Medical University, Shijiazhuang 050011, Hebei, China; 2 Postdoctoral Mobile Station, Hebei Medical University, Shijiazhuang 050017, Hebei, China; 3 Hebei Province Xingtai People’s Hospital Postdoctoral Workstation, Xingtai 054031, Hebei, China; 4 Department of Gynecology, Hebei Medical University Second Affiliated Hospital, Shijiazhuang 050000, Hebei, China; 5 Research Centre, The Fourth Hospital of Hebei Medical University, Shijiazhuang 050011, Hebei, China

**Keywords:** osteosarcoma, mesenchymal stem cells, leptin, autophagy, chemoresistance

## Abstract

Leptin signaling influences osteoblastogenesis and modulates the fate of mesenchymal stem cells (MSCs) during bone and cartilage regeneration. Although MSCs abound in the osteosarcoma (OS) microenvironment, and leptin exhibits pro-tumorigenic properties, leptin’s influence on OS progression and chemoresistant signaling in MSCs remains unclear. Using cell viability and apoptosis assays, we showed that medium conditioned by leptin-treated human MSCs promotes cisplatin resistance in cultured human OS cells. Moreover, GFP-LC3 expression and chloroquine treatment experiments showed that this effect is mediated by stimulation of autophagy in OS cells. TGF-β expression in MSCs was upregulated by leptin and suppressed by leptin receptor knockdown. Silencing TGF-β in MSCs also abolished OS cell chemoresistance induced by leptin-conditioned medium. Cisplatin resistance was also induced when leptin-conditioned MSCs were co-injected with MG-63 OS cells to generate subcutaneous xenografts in nude mice. Finally, we observed a significant correlation between autophagy-associated gene expression in OS clinical samples and patient prognosis. We conclude that leptin upregulates TGF-β in MSCs, which promotes autophagy-mediated chemoresistance in OS cells.

## INTRODUCTION

Osteosarcoma (OS) is the most common primary malignant bone neoplasm in children and adolescents [1]. Most cases are high-grade, and despite recent advances in therapeutic strategies combining chemotherapy, surgery, and sometimes radiotherapy, development of chemoresistance still compromises prognosis [2, 3]. Therefore, elucidating the specific mechanisms underlying chemotherapy resistance in OS is critical to improve patient outcomes.

Mesenchymal stem cells (MSCs) are nonhematopoietic fibroblast-like cells with potential for self-renewal, immune regulation, and multilineage differentiation [4–6]. MSCs are primarily found in the bone marrow but can also be derived from several other tissues. Interestingly, MSCs can migrate to tumor sites, interact with tumor cells, and become important constituents of the tumor microenvironment [7, 8]. There is substantial research supporting a role for MSCs in the growth, migration, and chemoresistance of OS cells [9–12]. Moreover, strong evidence suggests that OS cells may indeed originate from undifferentiated MSCs [13].

Leptin is primarily produced by fat tissue. It exerts anorexigenic effects, and its levels are typically increased in obesity, a condition associated with many chronic diseases, including diabetes, atherosclerosis, and cancer [4, 5]. However, leptin is also expressed in a variety of tissues, including placenta, ovaries, mammary epithelium, lymphoid tissues, and bone marrow, where it regulates multiple processes [6–8]. Notably, leptin signaling can regulate osteoblastogenesis, and has been implicated in the onset, progression, metastasis, and chemoresistance of different cancer types [14–16]. Our previous work demonstrated that leptin expression is associated with metastasis and poor prognosis in OS patients [17]; however, the specific mechanism(s) underlying leptin’s influence on OS malignancy remain to be defined. The leptin receptor is expressed in adult chondrocytes and osteocytes, and is also prominently expressed in a subset of MSCs, which stresses the important role of leptin on both bone formation and carcinogenesis through both direct and indirect effects [18–20]. In this work, we tested the hypothesis that leptin conditioning of MSCs promotes the survival of OS cells exposed to chemotherapy. Our findings suggest that targeting the leptin-TGF-β signaling axis in OS-associated MSCs may help overcome chemoresistance and improve patient prognosis.

## RESULTS

### Leptin conditioning of MSC promotes chemoresistance in OS cells

**Figure 1:**
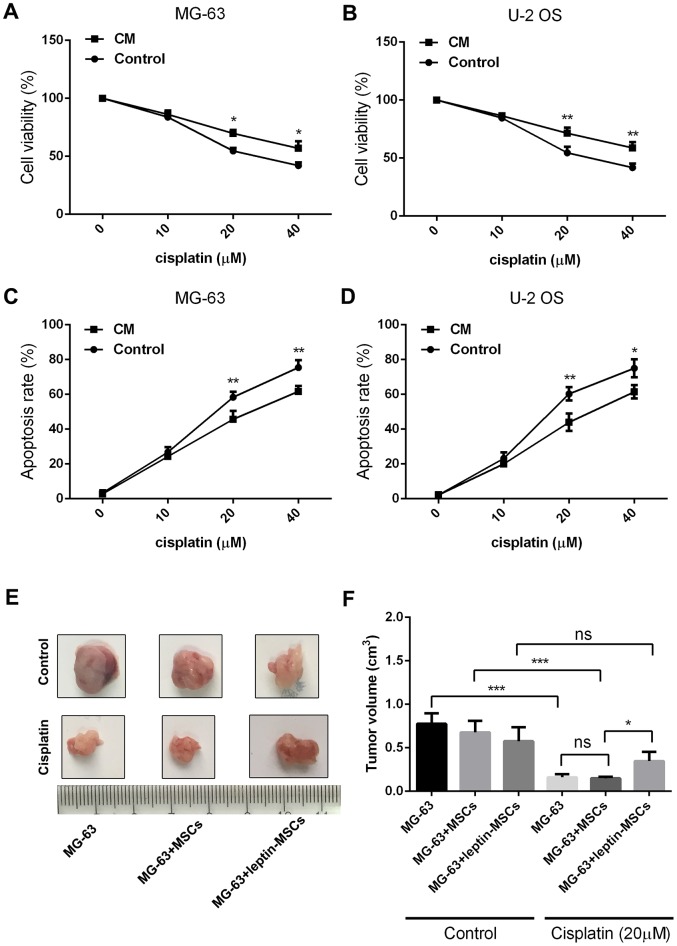
**CM from leptin-conditioned MSCs promotes chemoresistance in OS cells.** Results of CCK8 viability assays in cisplatin-treated MG-63 cells (**A**) and U-2 OS cells (**B**) incubated with CM collected from MSCs cultured in the presence (CM) or absence (Control) of leptin. *p<0.05; **p<0.01. Apoptosis was detected by PI/Annexin V-FITC flow cytometry in cisplatin-treated MG-63 cells (**C**) and U-2 OS cells (**D**) incubated with CM collected from MSCs cultured in the presence (CM) or absence (Control) of leptin. *p<0.05; **p<0.01. (**E**) Representative images of human OS xenografts excised from nude mice. (**F**) Quantification of tumor volumes at 21 days post-implantation. *p<0.05; ***p<0.001.

In a previous study we showed that high leptin levels and abundant numbers of MSCs characterize the osteosarcoma-associated microenvironment [17]. To test the hypothesis that leptin acts on MSCs to promote chemoresistance in OS, cultured human MSCs were treated with leptin (20 ng/ml) for 12 h, incubated in leptin- and serum-free DMEM for another 24 h, and the resulting conditioned media (CM) was added to cultured human OS cells (MG-63 and U-2 OS) in the presence or absence of cisplatin (0, 10, 20, or 40 μM). As shown in Figure 1A and 1B, a dose-dependent decrease in cell viability was recorded in cisplatin-exposed control cells incubated with CM from untreated MSCs, and this effect was significantly attenuated in OS cells treated with leptin CM. Using flow cytometry, apoptosis assays revealed a decreased apoptotic rate in cisplatin-treated OS cells incubated with leptin CM (Figure 1C and 1D). We next used a nude mouse model to evaluate whether leptin exposure could induce MSC-mediated chemoresistance in OS in vivo. In control conditions (no chemotherapy), final tumor volumes showed no differences between OS xenografts formed by MG-63 cells alone, MG-63 cells plus untreated MSCs, or MG-63 cells plus leptin-treated MSCs. However, after multiple intratumoral cisplatin injections, growth was unaffected only in tumors containing MG-63 cells plus leptin-treated MSCs (Figure 1E and 1F). These results indicated that leptin exposure promotes MSC-mediated chemoresistance in OS cells both in vitro and in vivo.

### Leptin-stimulated MSCs promote autophagy in OS cells

**Figure 2:**
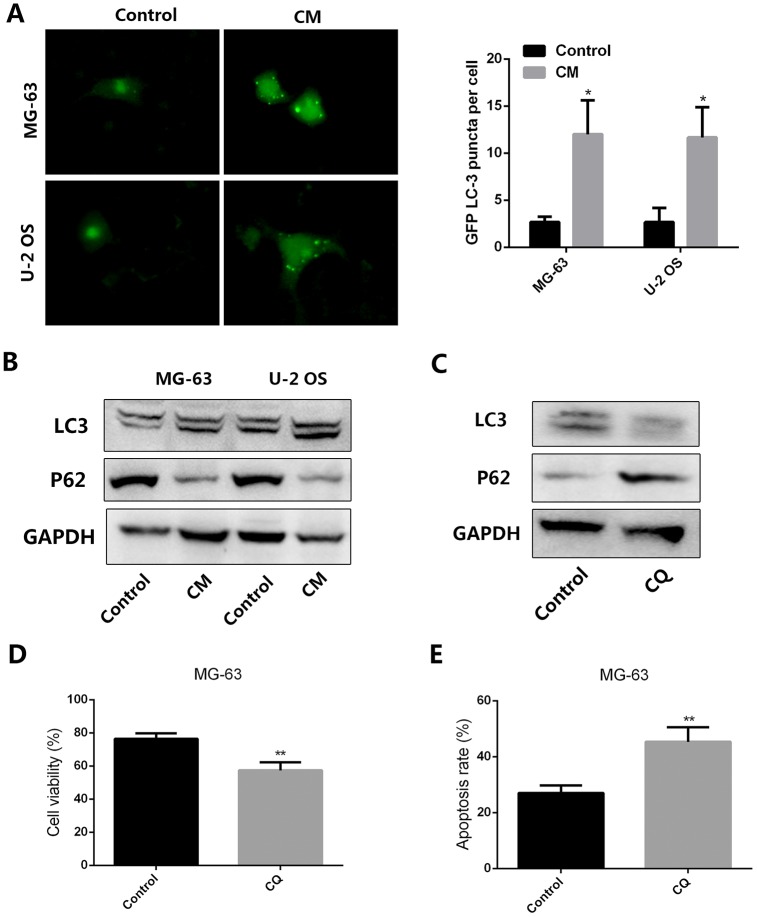
**CM from leptin-conditioned MSCs promotes autophagy in OS cells.** (**A**) Representative fluorescence images of cultured OS cells transfected with a GFP-LC3 vector and treated with CM collected from MSCs cultured in the presence (CM) or absence (Control) of leptin. Quantification of fluorescent puncta for each cell line is presented in the histogram shown on the right. *p<0.05. (**B**) Western blot analysis of LC3 and P62 expression in MG-63 and U-2 OS cells treated with CM from MSCs cultured in the presence (CM) or absence (Control) of leptin. (**C**) In the presence or absence of the autophagy inhibitor chloroquine (CQ, 10 μM), the expression of LC3 and P62 expression in MG-63 cells incubated with CM collected from leptin-conditioned MSCs was explored by western blot. (**D**) Effect of CQ (10 μM) on the viability of MG-63 cells exposed to cisplatin (40 μM) and CM from leptin-conditioned MSCs. **p<0.01. (**E**) Results of the PI/Annexin V-FITC flow cytometry assay showing the effect of CQ on apoptosis in MG-63 cells treated with cisplatin and CM from leptin-treated MSCs. **p<0.01.

Autophagy is an important process underlying tumor resistance to chemotherapy. To evaluate whether autophagy contributes to the chemoresistance of OS induced by leptin-stimulated MSCs, MG-63 and U-2 OS cells were transfected with a GFP-tagged LC3 vector. Results demonstrated that supplementation with CM from leptin-treated MSCs effectively increased punctate GFP fluorescence in cultured OS cells (Figure 2A). We also examined autophagy-associated gene expression using western blot. As shown in Figure 2B, addition of CM from leptin-treated MSCs to cultured OS cells led to significant upregulation of LC-3 and downregulation of P62 in the latter cells, indicating effective autophagy induction. We next examined whether autophagy inhibition using chloroquine (CQ) would attenuate the pro-survival effect of CM from leptin-conditioned MSCs on OS cells exposed to cisplatin. As shown in figure 2C, CQ effectively inhibited autophagy by decreasing LC3 and increasing P62 levels in MG-63 cells incubated with CM from leptin-conditioned MSCs. In line with these expression changes, CQ supplementation induced a significant reduction in OS cell viability, paralleled by an increased apoptotic rate, during concurrent cisplatin application (Figure 2D and 2E). These results indicate that autophagy is an important mechanism in the chemoresistance of OS cells induced by CM from leptin-exposed MSCs.

### Leptin increases TGF-β expression in MSCs

**Figure 3:**
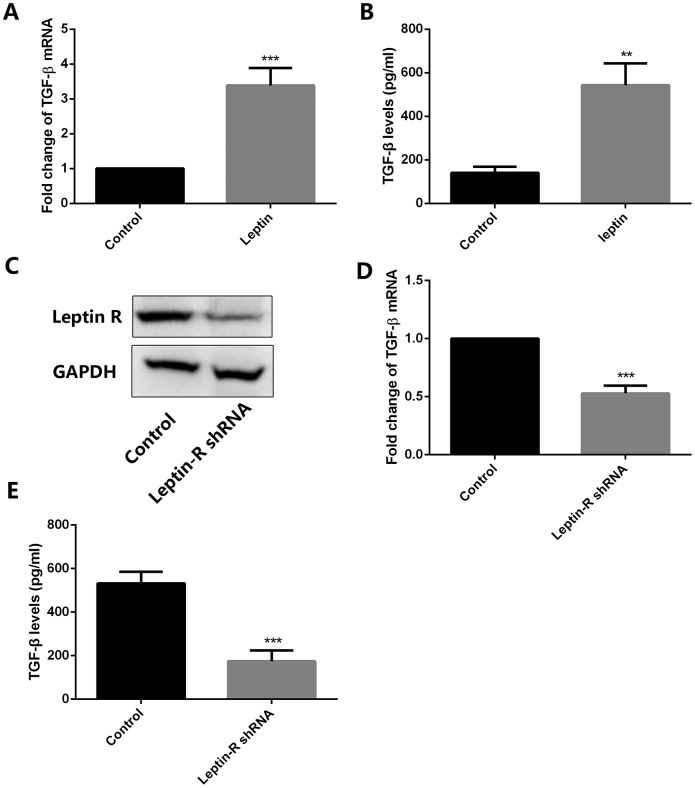
**Leptin stimulates TGF-β expression in MSCs.** (**A**) Analysis of TGF-β expression by RT-qPCR in control and leptin-treated MSCs. ***p<0.001. (**B**) ELISA detection of TGF-β levels in CM from control and leptin-treated MSCs. **p<0.01. (**C**) Leptin receptor (Leptin-R) expression was examined by western blot in both control and leptin-R shRNA-transfected MSCs. (**D**) TGF-β expression was tested by RT-qPCR in leptin-treated MSCs after leptin R knockdown. ***p<0.001. (**E**) TGF-β levels were examined by ELISA in control and leptin R-silenced MSCs exposed to leptin. ***p<0.001.

Since TGF-β has shown to induce autophagy in several tumor types [21, 22], we employed RT-qPCR and ELISA to examine its expression in both control and leptin-treated MSCs. Results showed that leptin significantly increased the expression and secretion of TGF-β in cultured MSCs (Figure 3A and 3B). To verify the direct role of leptin in the upregulation of TGF-β, we inhibited the expression of the leptin receptor (leptin-R) in MSCs using a specific shRNA (Figure 3C). As shown in Figure 3D and 3E, this procedure markedly inhibited leptin-R expression and significantly attenuated TGF-β expression in leptin-treated MSCs. These results indicated that leptin can directly enhance the expression of TGF-β in MSCs by acting on its receptor.

### TGF-β knockdown in MSCs decreases autophagy and chemoresistance in OS cells

**Figure 4:**
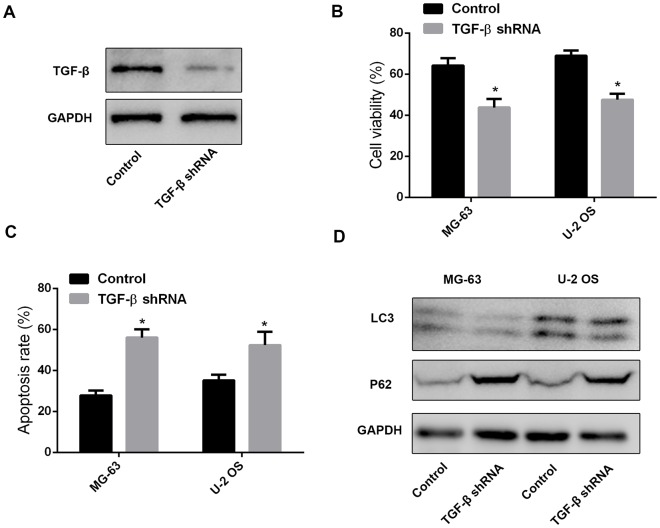
**MSC-derived TGF-β promotes chemoresistance and autophagy in OS cells.** (**A**) Western blot was performed to test TGF-β expression in leptin-treated MSCs after shRNA-mediated TGF-β knockdown. (**B**) CCK8 assay results from MG-63 and U-2 OS cells exposed to cisplatin (40 μM) in the presence of CM from leptin-treated MSCs transfected with TGF-β shRNA or a scrambled control shRNA. *p<0.05. (**C**) Apoptosis detection by PI/Annexin V-FITC flow cytometry in OS cells treated with CM from leptin- and TGF-β shRNA-treated MSCs. *p<0.05. (**D**) Western blot was employed to examine the expression of LC3 and P62 in OS cells treated with CM from leptin- and TGF-β shRNA-treated MSCs.

To verify that leptin-induced TGF-β expression in MSCs mediates autophagy and chemoresistance in OS cells, we transfected MSCs with a TGF-β-targeting shRNA. After confirming effective suppression of TGF-β expression (Figure 4A), cell viability and apoptosis analyses on OS cells treated with cisplatin revealed significant attenuation of chemoresistance and increased apoptosis after incubation with CM from leptin-treated, TGF-β-silenced MSCs (Figure 4B and 4C). Furthermore, western blot assays demonstrated that autophagy was effectively inhibited in OS cells treated with the above CM (Figure 4D).

### Clinical association between autophagy marker expression and OS prognosis

**Figure 5:**
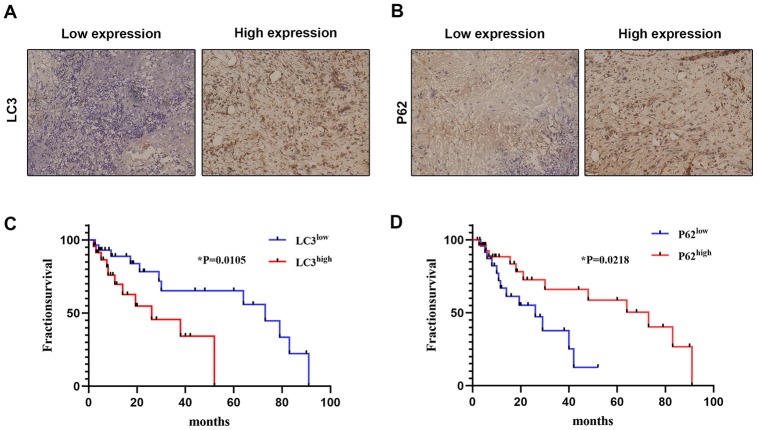
**Correlation between autophagy-related marker expression and OS prognosis.** The expression of LC3 (**A**) and P62 (**B**) was detected by IHC in clinical OS samples (×200). The correlation between OS survival rate and LC3 (**C**) and P62 (**D**) expression was analyzed using expression data from clinical OS specimens (Kaplan–Meier method); p values were obtained by log-rank multiple comparison tests.

Finally, we accessed clinical samples to explore the potential relationship between autophagy-associated genes (LC3 and P62) and prognosis in OS patients. Associations between LC3 and P62 expression and clinicopathological characteristics are summarized in Table 1. Representative images of LC3 and P62 staining in OS tissues are shown in Figure 5A and 5B. There were 24/54 cases with low LC3 expression and 30/54 cases with high LC3 expression. Meanwhile, 26/54 cases showed low P62 expression and 28/54 cases showed high p62 expression. Both LC3 and P62 expression levels were significantly associated with histologic grade (p<0.05), distant metastasis (p<0.05), and Enneking staging (p<0.05).

**Table 1:** Clinicopathological variables and the expression status of LC3 and P62 in OS patients.

**Characteristics**			**LC3**			**P62**		
			**Low (%)**	**High (%)**	**p**	**Low (%)**	**High (%)**	**p**
Gender	Female	23	9(39.1)	14(60.9)	0.498	13(41.9)	10(43.5)	0.289
	Male	31	15(48.4)	16(51.6)		13(56.5)	18(58.1)	
Age	<10 years	22	10(45.5)	12(54.5)	0.901	8(36.4)	14(63.6)	0.151
	>10 years	32	14(43.8)	18(56.3)		18(56.3)	14(43.8)	
Tumor size	<5 cm	24	13(54.2)	11(45.8)	0.198	8(33.3)	16(66.7)	0.051
	>5cm	30	11(36.7)	19(63.3)		18(60.0)	12(40.0)	
Histologic grade*	Well differentiated	15	13(86.7)	2(13.3)	<0.001	2(13.3)	13(86.7)	0.005
	Moderately differentiated	20	8(40.0)	12(60.0)		11(55.0)	9(45.0)	
	Poorly differentiated	19	3(15.8)	16(84.2)		13(68.4)	6(31.6)	
Distant metastasis*	No	30	19(63.3)	11(36.7)	0.002	10(33.3)	20(66.7)	0.015
	Yes	24	5(20.8)	19(79.2)		16(66.7)	8(33.3)	
Enneking stage*	I	10	8(80.0)	2(20.0)	0.001	2(15.4)	11(84.6)	0.002
	II	18	11(61.1)	7(38.9)		8(40.0)	12(60.0)	
	III	26	5(19.2)	21(80.8)		16(76.2)	5(23.8)	

* P < 0.05.

We also analyzed the correlation between the expression of LC3 and P62 and survival of OS patients. As shown in Figure 5C and 5D, high LC3 expression and low P62 expression were both significantly correlated with worse survival. These results suggest that the expression of autophagy markers such as LC3 and P62 may have prognostic value for OS patients.

## DISCUSSION

MSCs contribute to the occurrence and development of several tumors and are one of the most important cellular components of the OS microenvironment [10–12]. Research indicated that the receptor for leptin, an adipocyte-derived anorexigenic hormone with cytokine-like properties, is highly expressed in MSC and that leptin not only impacts the differentiation potential of MSCs but may also play an active role in carcinogenesis [14, 23–25]. In this work we found that CM collected from human MSC cultures treated with leptin effectively enhanced the resistance of human OS cell lines to the chemotherapy agent cisplatin. We further showed that this effect was secondary to stimulation of autophagy, a prominent tumor survival mechanism. The expression of TGF-β in MSCs was significantly upregulated by leptin and blocked by shRNA-mediated suppression of leptin receptor expression. In turn, TGF-β knockdown in MSCs markedly reduced cisplatin resistance in OS cells incubated with CM from leptin-treated MSCs. Moreover, the ability of MSCs to induce chemoresistance in OS cells was further confirmed by admixing leptin-treated MSCs and OS cells to generate xenografts in nude mice. Finally, we observed a significant correlation between autophagy-associated gene expression and the prognosis of OS patients.

Leptin can be detected in the microenvironment of several tumors, including breast and lung cancers, where it contributes to an aggressive tumor phenotype by inducing cell growth, migration, and invasion [26–29]. Leptin is also produced by osteoblasts, and its levels are associated with metastasis and poor prognosis in OS patients [30]. Our previous clinical study also indicated that high levels of leptin correlated with poorer prognosis in OS patients [17], however, the underlying mechanisms remained so far unclear. In this regard, the present data strongly suggest that the tumor-promoting capacity of leptin, and its facilitatory role in the onset of chemoresistance, are linked to its ability to promote autophagy in OS cells by upregulating TGF-β in tumor-associated MSCs.

Autophagy is associated with several physiological and pathological processes such as cell differentiation and stress survival, tumorigenesis, and tumor therapy resistance [31]. Autophagy is indeed a key mechanism for cell survival in harsh microenvironments like the hypoxic tumor microenvironment, by sustaining cell growth and cellular homeostasis [32–34]. Several factors, including hypoxia, starvation, and TGF-β signaling, are capable of inducing autophagy [35–37]. LC3 and P62 are the most widely used autophagy markers. LC3-I is found in the cytoplasm and upon lipidation into LC3-II is incorporated to the autophagosomal membrane [38]. P62, also known as sequestosome 1 (SQSTM1), functions as an autophagy receptor and is involved in autophagy-dependent elimination of many different cargos, including ubiquitinated protein aggregates and bacteria. P62 is in turn constantly degraded by autophagy, as the autophagic flux progresses [39]. Our results showed that exposure of OS cells to CM from leptin-conditioned MSCs led to upregulation of LC3 and downregulation of P62, which indicated the occurrence of autophagy.

TGF-β is a multifunctional regulator of cell growth, differentiation, and migration, and autocrine TGF-β signaling importantly affects several MSC functions [40]. TGF-β enhances the expression of autophagy-associated genes, including Atg5, Atg7, and Beclin1, and induces also the conversion of LC3-I into LC3-II, leading to the formation of autophagosomes. Accordingly, TGF-β was shown to promote autophagy in several tumor types such as mammary carcinoma cells and glioblastoma [21, 41, 42]. In line with those findings, our results demonstrated that inhibition of TGF-β secretion by leptin-treated MSCs decreased autophagy in OS cells, as indicated by reduced LC3 and increased P62 expression. In summary, our results indicate that leptin signaling upregulates TGF-β in MSCs, which promotes autophagy-mediated chemoresistance in OS cells. These findings may aid the design of new therapeutic strategies that target the leptin-TGF-β axis in the OS microenvironment.

## MATERIALS AND METHODS

### Reagents

Cisplatin was purchased from Qilu Pharmaceutical Co., Ltd. (Jinan, Shandong, China; Cat.H20073653). Leptin was purchased from PeproTech (La Jolla, CA, USA; Cat. 300-02B,). Chloroquine (CQ, Cat.C6628) was purchased from Sigma-Aldrich (St. Louis, MO, USA).

### Cell culture

Human umbilical cord mesenchymal stem cells were purchased from Cyagen Biotechnology Co., Ltd. (Cat. HUXUC-01001) and were cultured in Dulbecco’s modified Eagle’s medium (DMEM) nutrient mix F12 with 10% fetal bovine serum (FBS, Cat. 10099-054; Invitrogen, Corp., Carlsbad, CA, USA). Human osteosarcoma (OS) cell lines MG-63 and U-2 OS were purchased from the Chinese Cell Bank of the Chinese Academy of Sciences (Shanghai, China) and cultured in DMEM supplemented with 10% FBS (Life Technologies/Thermo Fisher Scientific, Beijing, China), streptomycin (100 U/mL), and penicillin (100 U/mL) at 37°C in a humidified atmosphere containing 5% CO_2_.

### Preparation of leptin-conditioned media

Leptin (20 ng/ml) was used to treat MSCs for 12 h. Then the culture medium was replaced by FBS-free DMEM and cells were cultured for another 24 h. The resulting conditioned medium was then collected and filtered through a 0.22 μm filter.

### RNA extraction and real-time PCR

Total RNA was extracted from cells using TRIZOL (Invitrogen) and cDNA synthesis was performed using the PrimeScript RT reagent Kit (Takara, Kyoto, Japan) according to the manufacturer’s instructions. TGF-β mRNA was quantified by real-time quantitative PCR. TGF-β mRNA primers were: 5’-GCCGAGCCCTGGACACCAAC-3’ (forward) and 5’-GCGCCCGGGTTATGCTGGTT-3’ (reverse). PCR was performed using the SYBR Green PCR Kit (Applied Biosystems) according to the manufacturer’s instructions. GAPDH was used as internal reference.

### Cell viability assay

OS cells (1×10^4^/well) were seeded into 96-well plates in complete medium (200 μL). After incubation for 24 h at 37°C/5% CO_2_, the medium was replaced by complete medium containing cisplatin (0 to 40 μM) along with MSC-derived CM. After an additional 24 h, cell viability was examined with the CCK8 assay, and optical density (OD) was determined at 490 nm using a microplate reader.

### Apoptosis assay

After experimental treatments, cultured OS cells were harvested into single-cell suspensions and apoptosis was detected by flow cytometry using an Annexin V-FITC/PI apoptosis detection kit (KeyGen, Nanjing, China) according to the manufacturer’s protocol. Results are expressed as the percentage of apoptotic (PI-negative/Annexin V-positive) cells in the gated cell population.

### Cell transfection

A GFP-tagged LC3 expression vector was used to detect autophagy. OS cells were plated into 96-well plates, cultured to 70% confluence, and GFP-LC3 expressing plasmids were transiently transfected using FuGene HD transfection reagent (Roche, NSW, Australia, Cat.04709705001). Autophagy induction was evaluated by counting the number of OS cells with GFP-positive dots by fluorescence microscopy.

The TGF-β shRNA and control shRNA sequence were 5’-ACCAGAAATACAGCAACAATTCCTG-3’ (shTGF-β) and 5’-GTACCTGACATGCACTTCCAATGAC-3’ (shCon), respectively. pGCL-GFP lentiviral particles encoded GFP and shRNAs. Cells (1-3×10^6^) growing to 50%-60% confluence in 10 cm petri dishes were transfected with lentiviral, and western blot was used to examine the targeted gene expression after lentiviral transfection.

### In vivo tumorigenesis assay

MSCs (1 × 10^5^) were treated with or without leptin (20 ng/ml) for 12 h and admixed with MG-63 cells (5 × 10^5^) prior to subcutaneous implantation into the armpit areas of male Balb/C nude mice (n=7 per group). Control animals (n=7 per group) were implanted with MG-63 cells alone. When the tumors become palpable, cisplatin (4 mg/kg) or saline as control vehicle was injected into the tumors every 3 days. The mice were sacrificed, and tumors removed 21 days following implantation. Tumor growth was evaluated by measuring the length and width of the tumor mass. All animal experiments were performed in accordance with the Institutional Animal Welfare Guidelines of Hebei Medical University.

### TGF-β1 measurement

TGF-β1 levels in CM from MSC cultures were determined using an ELISA kit (R&D Systems, USA) according to manufacturer’s instructions.

### Western blot analysis

Whole-cell protein lysates were extracted using ProteoJET Mammalian Cell Lysis Reagent (MBI Fermentas, Canada) supplemented with complete protease inhibitor cocktail according to the manufacturer’s instructions. Protein concentration was determined using the Bradford protein assay kit (Beyotime Institute of Biotechnology, China). Protein samples were separated on a 10% SDS-PAGE gel and transferred onto nitrocellulose membranes (Millipore, USA). The membranes were blocked in PBS containing 5% skimmed milk and Tween-20 for 2 h and incubated overnight at 4°C with primary antibodies against LC3 (Abcam, 1:500), P62 (Abcam, 1:1000), leptin-R (Abcam, 1:1000), TGF-β (Abcam, 1:200), and GAPDH. Horseradish peroxidase-conjugated secondary antibodies were next applied for 30 min at 4 °C. Protein bands were visualized with SuperSignal chemiluminescent substrate (Thermo Scientific, USA). GAPDH was used as a loading control.

### Immunohistochemistry

Tissue samples were fixed in 4% paraformaldehyde and embedded in paraffin. Anti-LC3 (Abcam, 1:500) and Anti-P62 (Abcam, 1:1000) were used as primary antibodies. Immunohistochemical staining was carried out using a previously described protocol [17, 43]. A total of 54 OS sections were collected from patients at the Fourth Hospital of Hebei Medical University (Shijiazhuang, China).

### Statistical analysis

All data were analyzed using SPSS 19.0 statistical software. Data were analyzed using one-way ANOVA or Student’s t-test, and values are presented as the mean ± standard deviation (SD). Each experiment was carried out at least three times, and P < 0.05 was considered significant.
